# Thermodynamics Controlled Sharp Transformation from InP to GaP Nanowires via Introducing Trace Amount of Gallium

**DOI:** 10.1186/s11671-021-03505-2

**Published:** 2021-03-20

**Authors:** Zhenzhen Tian, Xiaoming Yuan, Ziran Zhang, Wuao Jia, Jian Zhou, Han Huang, Jianqiao Meng, Jun He, Yong Du

**Affiliations:** 1 Hunan Key Laboratory of Super Micro-structure and Ultrafast Process, School of Physics and Electronics, Central South University, Changsha, 410083 China; 2 College of Mechanical and Vehicle Engineering, Hunan University, Changsha, 410082 China; 3 State Key Laboratory of Powder Metallurgy, Central South University, Changsha, 410083 China

**Keywords:** Nanowire growth, GaP, InP, Chemical vapor deposition, CALPHAD

## Abstract

Growth of high-quality III–V nanowires at a low cost for optoelectronic and electronic applications is a long-term pursuit of research. Still, controlled synthesis of III–V nanowires using chemical vapor deposition method is challenge and lack theory guidance. Here, we show the growth of InP and GaP nanowires in a large area with a high density using a vacuum chemical vapor deposition method. It is revealed that high growth temperature is required to avoid oxide formation and increase the crystal purity of InP nanowires. Introduction of a small amount of Ga into the reactor leads to the formation of GaP nanowires instead of ternary InGaP nanowires. Thermodynamic calculation within the calculation of phase diagrams (CALPHAD) approach is applied to explain this novel growth phenomenon. Composition and driving force calculations of the solidification process demonstrate that only 1 at.% of Ga in the catalyst is enough to tune the nanowire formation from InP to GaP, since GaP nucleation shows a much larger driving force. The combined thermodynamic studies together with III–V nanowire growth studies provide an excellent example to guide the nanowire growth.

**Supplementary Information:**

The online version contains supplementary material available at 10.1186/s11671-021-03505-2.

## Introduction

Nanowires show advantages in strain relaxation, heterojunction formation as well as crystal phase engineering and thus are developing rapidly during the last decade [1–4]. III–V semiconductor nanowires, thanks to their superior optical and electronic properties, have been widely used in both application fields (such as photovoltaics [5], photodetectors [6, 7], photodiodes [8], and electronic devices [9]) and basic science researches [10]. For the bottom-up nanowire fabrication, metal organic chemical vapor deposition (MOCVD) [11, 12] and molecular beam epitaxy (MBE) [13] techniques are widely applied to synthesize high-quality nanowires. For instance, both pure wurtzite [14] and zincblende twinning superlattice InP nanowires [15, 16] have been demonstrated and applied in terahertz detection [17] and lasing applications [18]. However, these high-quality nanowire is produced at a high cost. Instead, using cheap chemical vapor deposition (CVD) method can largely reduce the production costs for III–V nanowires and expand their application fields, such as photoelectrochemical field [19]. Moreover, CVD owns advantages in growth exploration thanks its feasibility [20]. III-P and their ternary InGaP nanowires, thanks to their suitable bandgap, low toxicity as well as low surface recombination velocity [14], thus showing potential in biosensors [21], lasers [22], and photocatalysis [23] applications. Hui et al. [24] demonstrated CVD growth of InP nanowires with a high mobility (~ 350 cm^2^ V^−1^ s^−1^) comparable to nanowires grown by MOCVD and MBE technologies. Using InP nanowire fabricated via CVD method, Zheng et al. [25] fabricated ferroelectric polymer side-gated single InP NW photodetectors, showing an ultra-sensitive photodetection in which the dark current is greatly suppressed by the local electric field generated by this ferroelectric material. GaP is a semiconductor with band gap of 2.26 eV and high refractive index and thus is a good candidate for light-emitting diodes in green-yellow region [26] as well as photonics applications [27]. Moreover, the suitable bandgap of GaP makes it also useful in the field of photocatalysis [23, 28]. But still, reports of CVD growth of GaP and its ternary InGaP nanowires are quite limited. GaP nanowires were mainly produced via physical vapor deposition method [18, 20, 29, 30]. Ternary InGaP nanowires were demonstrated by MOCVD [31, 32], MBE [33] as well as solution phase synthesis method [23]. The detailed growth, as well as growth fundamentals of metal-seeded GaP and InGaP nanowires, need further exploration. Thermodynamics is a significant factor that affects nanowire growth. CALPHAD is a powerful and well-established method to calculate the phase equilibria and thermodynamic properties of bulk materials [34]. This semiempirical thermodynamic calculation method can calculate the thermodynamic properties during nucleation, thus guiding nanowire growth. CALPHAD method has been applied to calculate the nanophase diagram of In–Sb system [35] and understand the Au-seeded growth of GaAs and InAs nanowires [36] as well as composition analysis in InGaAs nanowires [37]. Still, there is much work to be done to fully apply CALPHAD method to guide the III–V nanowire growth. For instance, no CALPHAD analysis has been performed to explain the growth behavior of the Au-seeded InGaP nanowires.

In this work, using InP powder and metal Ga as precursors, a high density of InP and GaP nanowires is grown in a CVD reactor under vacuum conditions. This method is demonstrated to be effective in producing nanowires in a wide temperature range. After optimizing the InP nanowire growth, we further investigate the growth of GaP nanowires by introducing pure Ga into the reactor. Instead of forming ternary InGaP nanowires, nearly pure GaP nanowires are formed independently of the input weight of Ga or the growth temperature. Further composition determinations and thermodynamic calculations show that the nanowire composition is controlled by thermodynamics instead of kinetics. A small content of Ga in the Au droplet can tune nanowire growth from InP to GaP, well explaining the observed nanowire growth behavior. This work provides a low-cost and effective method for III–V nanowire growth, and the applied phase diagram analysis method is valuable for understanding the III–V nanowire growth.

## Methods

### Preparation of InP and GaP Nanowires

**Fig. 1:**
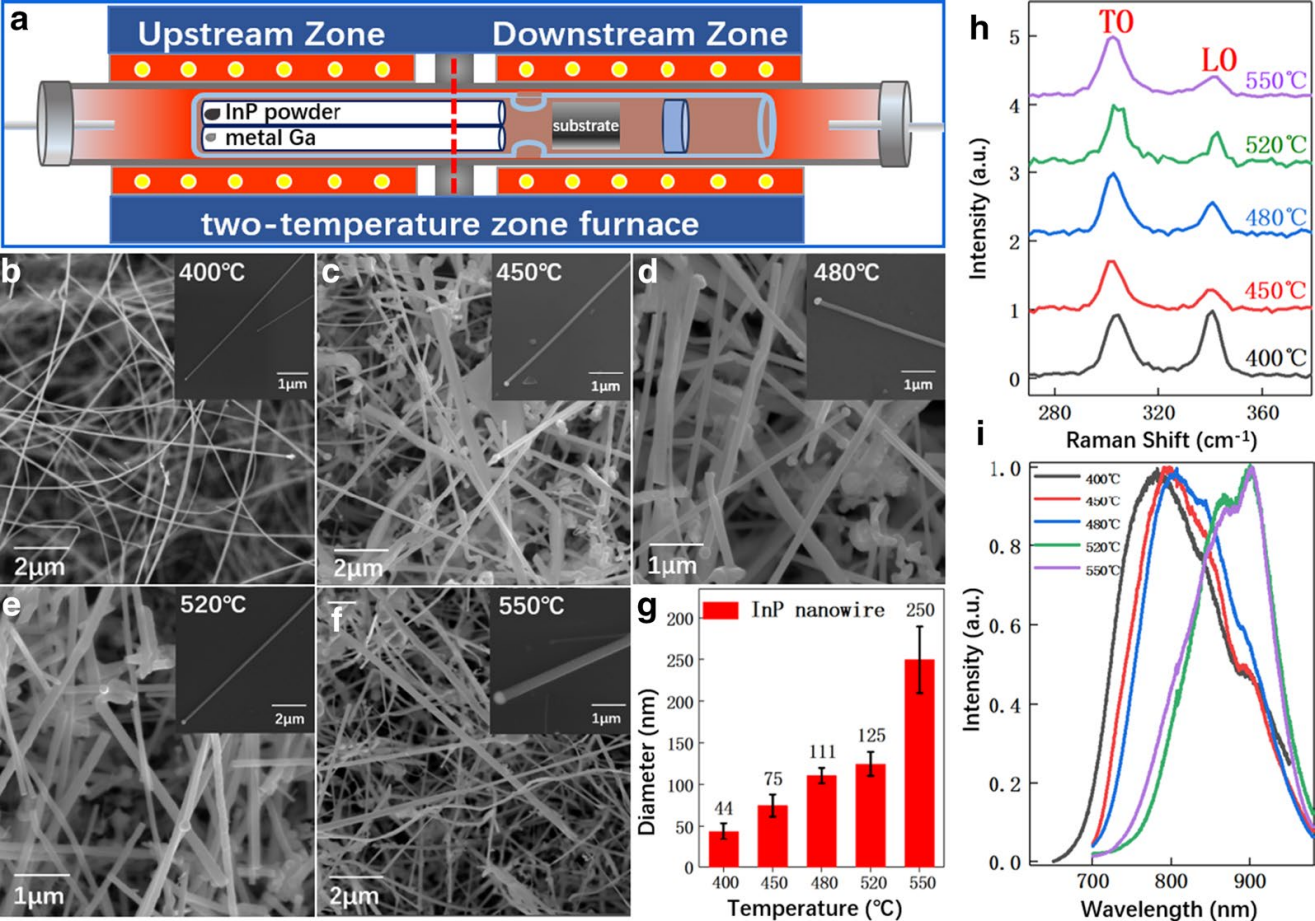
Growth studies of InP nanowires. **a** Schematic illustration of the experimental setup for both InP and GaP nanowires growth. SEM images of InP nanowires grown at **b** 400 °C, **c** 450 °C, **d** 480 °C, **e** 520 °C and **f** 550 °C. **g** Diameter distribution of nanowires prepared at different temperatures. **h** Raman and **i** PL comparison of nanowires grown at different temperatures

InP and GaP nanowires were grown in a home-build CVD system under vacuum conditions, as illustrated in Fig. 1a. Highly purified Ga (99.999%, Innochem) and InP powders (99.99%, Aladdin) were used as solid resources and separated into two isolated quarts tubes. The inner diameter of quartz tubes is 8 mm with a length of 180 mm. Around 2-nm-thick Au film was deposited on the Si(111) substrate using e-beam evaporation. These quartz tubes, together with Au deposited Si(111) substrate, were loaded inside another large quartz tube (as illustrated in Fig. 1a) and sealed by a vacuum sealing machine (Partulab MRVS-1002). The pressure of the whole tube is ~ 3.0 × 10^–3^ Pa. Then, sample growth was performed in a two-temperature zone furnace. The temperature of the first zone and InP powder weight were kept constant for all the samples at 720 °C and 20 mg, respectively. For InP nanowire growth, no Ga powder was introduced and the second growth temperature zone was varied from 400 to 550 °C. After InP nanowire growth optimizations, Ga power (0–5 mg) was added to grow InGaP nanowire in a temperature range from 520 to 630 °C. During the temperature-dependent growth, the Ga weight was fixed at 3 mg. Raise the temperature zone to the targeted temperature, keep the temperature for 60 min, and then cool down.

### Nanowire Characterizations

After growth, the morphology and structure of the nanowires were investigated by scanning electron microscope (SEM) and transmission electron microscope (TEM) operated at 300 kV (Titan G2 60-300). The crystal structure of as-grown nanowires was investigated by X-ray diffraction (MiniFle × 600). For detailed composition measurements, energy-dispersive spectrum (EDS) equipped in both SEM and TEM was applied. Thermo-Calc software was used to perform thermodynamic calculations. The optical properties of InP and GaP nanowires were examined by micro-Raman and photoluminescence (PL) in a commercial Renishaw system (inVia). In short, nanowires were pumped via a green laser (532 nm) through an objective lens (100 ×).

## Results and Discussions

### InP Nanowires

After growth, a high dense of InP nanowires could be observed under optical microscopy for all the investigated growth temperature ranges of 400–520 °C. Detailed morphology characterization in Fig. 1b–g shows the non-vertical and randomly distributed InP nanowires on the Si(111) substrate, which is similar to other nanowires grown by CVD [20]. In general, all the nanowires are over 10 μm long with nearly taper-free morphology, which is much longer than the III–V nanowire growth rate by MBE [38] or MOCVD [39]. The enlarged SEM images in the insets show the morphology of a single nanowire. Au droplet is observed at the growth front, indicating that the InP nanowire growth is controlled by the well-known vapor–liquid–solid (VLS) growth mechanism [11] In addition to inclined and curved nanowires, in-plane InP nanowires are also observed on the substrate (see the insets in Fig. 1). Despite the morphology variation, it seems that growth temperature affects the nanowire diameter. At low growth temperature (400 °C), the nanowire is relatively thin, with an average diameter of 121 nm. With the increase in growth temperature, nanowire diameter monotonously increases but distributes more disorderly. For instance, at 550 °C, nanowires with diameters from 210 to 290 nm are observed, and the distribution of nanowires on the silicon substrate is not uniform.

Raman scattering and PL techniques were used to quickly test the crystal quality and optical properties of the as-grown InP nanowires, as compared in Fig. 1h. Two peaks at ~ 302 cm^−1^ and 341 cm^−1^ are observed for all the samples, which correspond to the longitudinal optical (LO) and the traverse optical (TO) phonon modes of InP [40]. This suggests that all the fabricated nanowires are indeed InP. However, the corresponding PL data in Fig. 1i are quite confusing. For nanowires grown between 400 and 480 °C, PL spectra show a strong and broad emission peak in the range of ~ 775 nm to 811 nm. The emitted photon energy is much larger than the bandgap of either wurtzite (WZ) (872 nm) or zincblende (ZB) (922 nm) InP nanowires, suggesting that the emission is not from pure InP. The concave at around 886 nm is caused by a system bug in our optical system. When the temperature is above 520 °C, a strong emission peak around 900 nm is observed, which is ascribed to the emission from polycrystal InP nanowires [40]. These studies suggest that the optimal growth temperature for InP nanowires is ~ 520 °C, leading to a uniform distribution of InP nanowires with high optical quality.

**Fig. 2:**
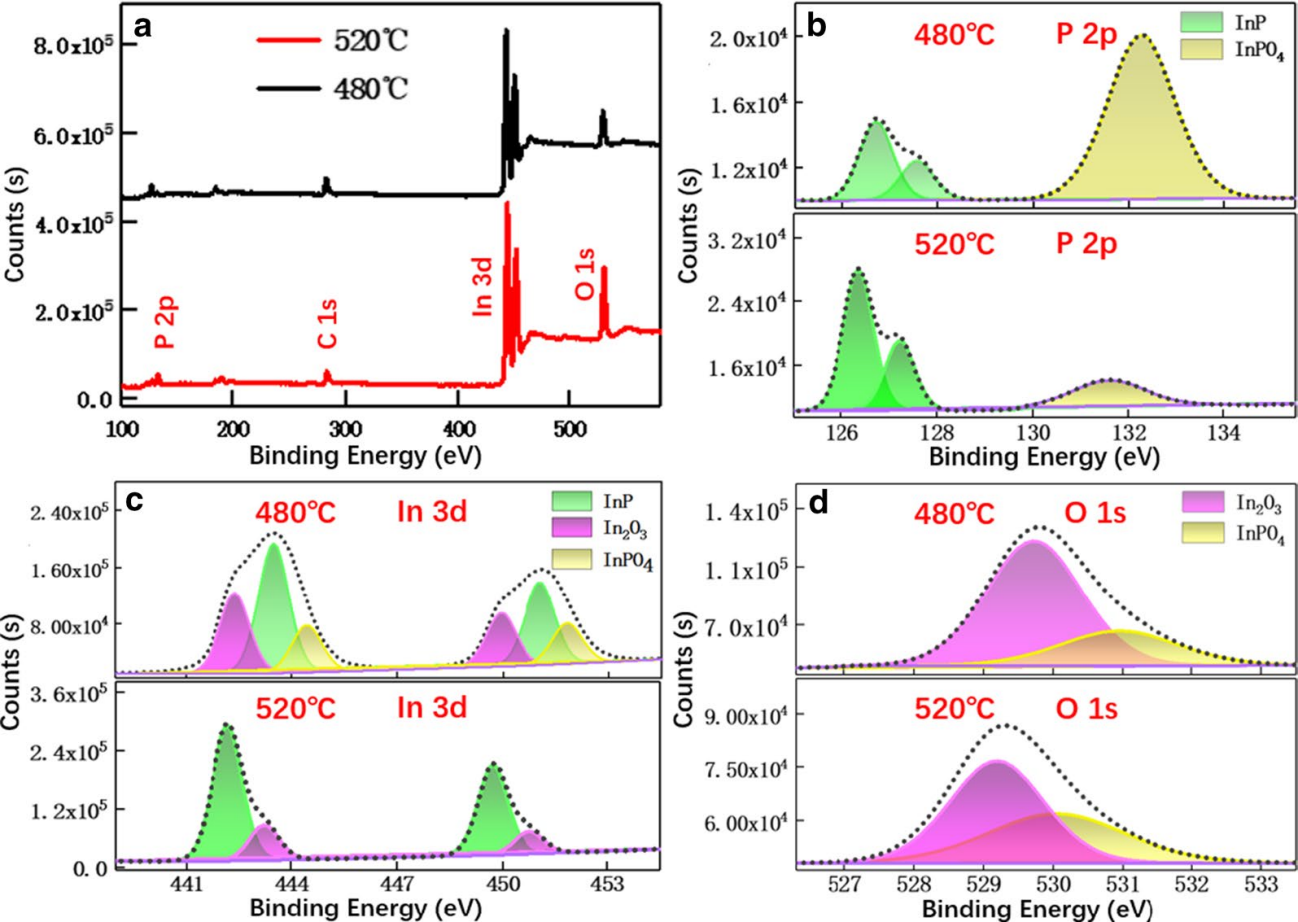
XPS comparison of InP nanowires surface grown at temperature 480 °C and 520 °C. **a** Survey spectrum, high-resolution XPS spectra of the **b** P-2*p*, **c** In-3*d*, **d** O-1*s*

To clarify the observed PL spectra difference, X-ray photoelectron spectroscopy (XPS) tests for samples grown at 480 and 520 °C were performed under the same test conditions, as compared in Fig. 2. For both samples, XPS spectra show characteristic peaks from In-3*d* and P-2*p*. In addition, O-1*s* and C-1*s*-related peaks were recorded as well. The slow scan results of In-3*d* peak of sample grown at 480 °C (see Fig. 2c) can be deconvolved into three peaks at 443.5, 442.3, and 444.4 eV, which are ascribed to InP, In_2_O_3,_ and InPO_4_ [41, 42], respectively. Based on the relative intensity, the weights ratio of the above compounds is 31.0%, 48.7%, and 20.3%, respectively. The strong P-2*p* peak at 132 eV (see Fig. 2b) further confirms the existence of InPO_4_. In comparison, for the sample grown at 520 °C, the peak intensity of In-3*d*, P-2*p*, and O-1*s*, representing InPO_4_, In_2_O_3_, is largely suppressed while the relative intensity for InP is enhanced. These comparisons demonstrate that higher growth temperature is able to suppress the oxide formation and increase the purity of InP. At lower growth temperature, the oxide formation in InP nanowire cannot be ignored and the PL emission is dominated by the indium oxide, thus showing a broad emission peak caused by In_2_O_3_ defect state [43, 44]. Instead, the increased purity of InP nanowires at higher growth temperature leads to the characteristic peak from InP semiconductor. Also, these experiments indicate that in addition to the growth condition itself, the experimental process should be careful to avoid the introduction of oxygen in the sealed tube. For instance, the vacuum should be even higher to avoid oxygen content. Moreover, during the sealing process, the InP powder should be cooled to avoid possible oxidation.

**Fig. 3:**
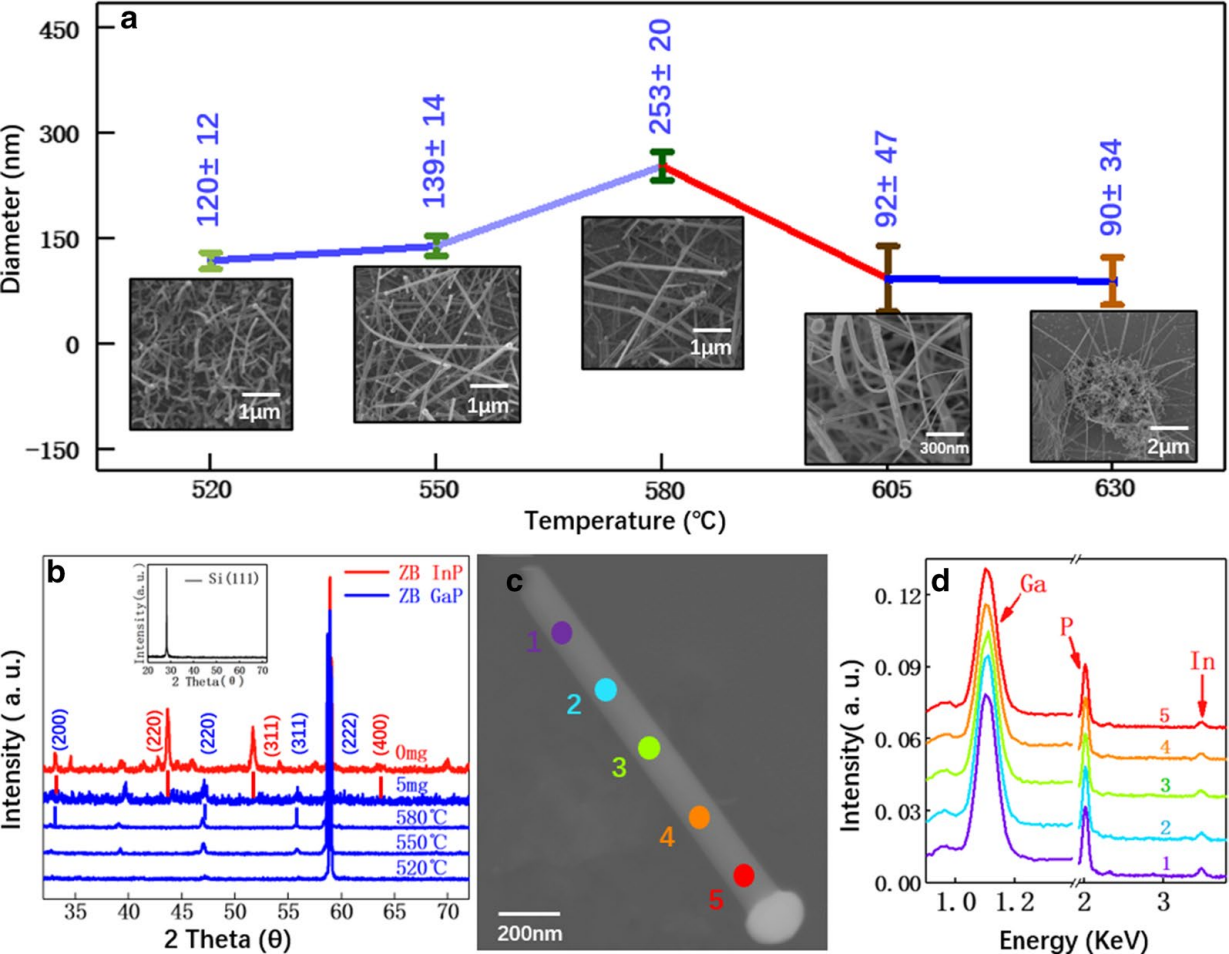
Synthesis of InGaP nanowires. **a** Diameter distribution of InGaP nanowires at different growth temperatures (550–630 °C) with embedded characteristic SEM images. **b** XRD spectra of InP (red curve) and InGaP (blue curves) nanowires at different growth conditions. XRD spectrum of Si(111) substrate is shown in the inset as a reference. SEM (**c**) and the corresponding EDS spectra (**d**) of a InGaP nanowire grown at 550 °C. The gallium powder weight is 3.0 mg

After InP nanowire growth studies, Ga power (3 mg) was introduced into the reactor to grow ternary InGaP nanowires. The addition of Ga leads to a high density of nanowire formation in the temperature range from 520 to 630 °C. The substrate even turns to yellow color. The average nanowire diameter increases from 90 to 253 nm before reducing again after 580 °C (see Fig. 3a). The crystal and composition of nanowires grown at different conditions are first examined by XRD, as compared in Fig. 3b. The used Si(111) substrate shows only one main peak at 28.43°. For InP nanowires grown at 550 °C, additional peaks at 33.08°, 43.61°, 51.71°, 58.93°, and 63.52° are observed and are ascribed to (200), (220), (311), (222), (400) planes of ZB InP [45]. For the InGaP nanowires, the XRD spectra of nanowires grown at all the investigated conditions (either temperature-dependent or Ga weight dependent) are quite similar with nearly the same peak position, peaking at 32.64°, 46.93°, 55.80°, and 58.93°. These peaks represent (200), (220), (311), and (222) planes of ZB GaP [46]. Even though the input weight ratio of InP and Ga powder represents a nominal composition of In_0.44_Ga_0.56_P, XRD data suggest the successful growth of GaP nanowires instead of the expected ternary InGaP nanowires. This is quite interesting since only a small amount of Ga powder is able to fully convert the nanowire growth from InP to GaP. For an accurate investigation of this phenomenon, we transfer these nanowires to the Si substrate for energy-dispersive X-ray spectroscopy (EDX) analysis. Typical SEM image and corresponding EDX spectra of a nanowire grown at 550 °C with Ga powder of 3.0 mg are shown in Fig. 3c, d. The EDX spectra show only dominant peaks from Ga and P with only a very weak peak of In. Moreover, EDX analysis along this nanowire shows the same composition distribution. This conclusion is valid for all the measured nanowires. These EDX spectra are in good agreement with the XRD results that the as-grown nanowires are mainly GaP.

**Fig. 4:**
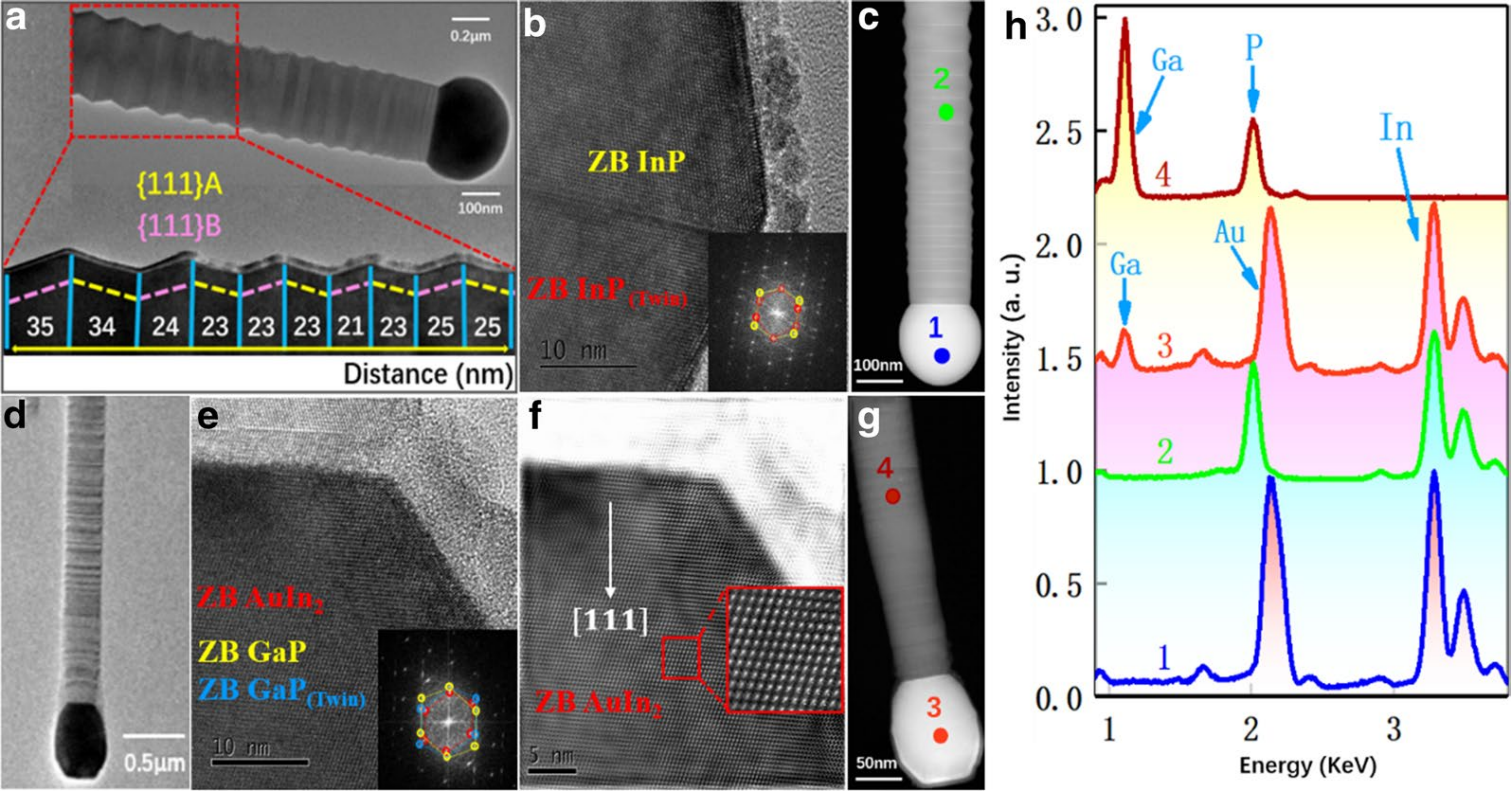
Structural and composition analysis of InP and GaP nanowires. **a**, **b** HRTEM image of an InP nanowire, showing the ZB twinning superlattice structure. **d**, **e** TEM images of a GaP nanowire. **f** Fast Fourier transform image of the Au droplet, demonstrating the formation of AuIn_2_ phase. High-angle annular dark-field (HAADF) image of the same InP (**c**) and GaP (**g**) nanowires. **h** EDX spectra of points 1–4 in (**c**, **g**). EDX intensity is normalized and shifted for visibility

To further reveal the growth fundamental, TEM analysis of InP and GaP nanowires is performed and shown in Fig. 4. Commonly the main InP and GaP nanowires are grown along the [111] direction with ZB structure [47, 48], agreeing well with the above XRD analysis. In particular, InP nanowires present a twinning superlattice like structure (see Fig. 4a), which is similar to the InP TSL nanowires grown at high temperature via MOCVD [16]. The periodic twin plane distance slightly fluctuates between 35 and 21 nm and seems to decrease along the growth directions, especially close to the Au droplet. In comparison, a high density of planar defects is found in GaP nanowires. High-resolution TEM (HRTEM) image close to the Au droplet (see Fig. 4e, f) shows that the droplet mainly consists of AuIn_2_ with ZB phase [49]. Moreover, AuIn_2_ and GaP nanowires present the same crystal orientation. Thus, it suggests that AuIn_2_ phase is epitaxially grown on the GaP nanowire during the solidification process. The Au droplet on InP nanowire shows the same brightness contrast, suggesting a single phase. In comparison, it seems that a small amount of Au-rich layer is formed after the solidification of AuIn_2_ based on the brightness contrast in Fig. 4g as well as EDX mapping in Figure S1 in the Additional file 1. EDX analysis comparison in Fig. 4h confirms the formation of GaP nanowires, and nearly, no In peak is observed. However, indium is the major element in the catalyst. The relative ratio between In and Au is the same for both InP and GaP nanowires. Based on the HRTEM analysis in Fig. 4e, the catalyst phase is mainly of AuIn_2_. The introduction of Ga does not reduce the In content but only leads to a small concentration of Ga in the catalyst. However, the content of Ga is high enough to inhibit nucleation of In from the droplet into the nanowire, thus only forming GaP nanowires. The large catalyst shape difference in InP and GaP nanowires is caused by the local surface tension differences [50]. These EDX observation raises a question that why a much higher In content in the catalyst does not lead to the formation of In-rich InGaP nanowires.

**Fig. 5:**
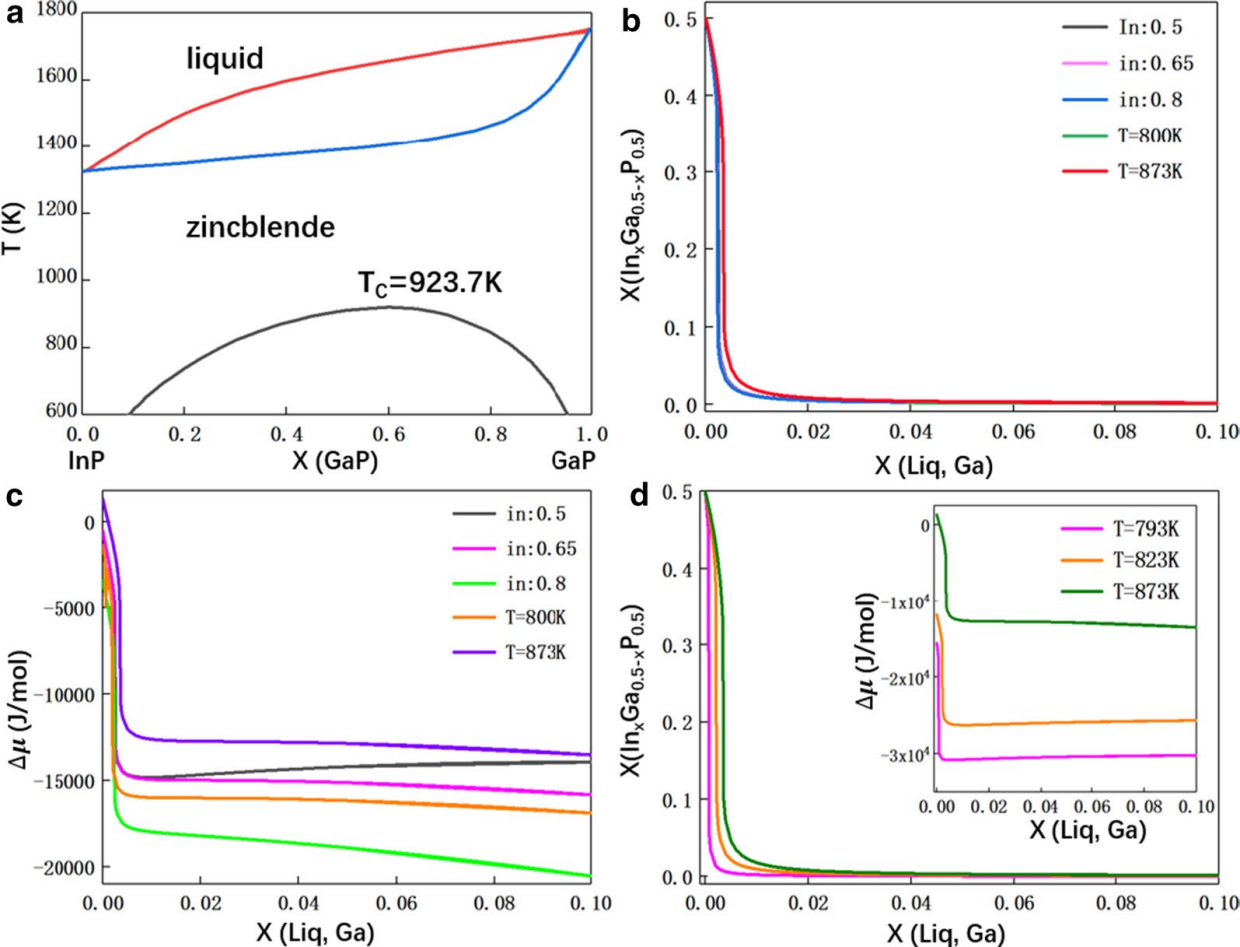
Thermodynamic analysis of the nucleation process. **a** Pseudo-binary InP-GaP phase diagram. Calculated (**b**) In content in In_*x*_Ga_0.5-*x*_P_0.5_ and Gibbs energy change (**c**) as a function of Ga content in the Au droplet. **d** Composition and Gibbs energy analysis for InGaP nanowire formation under In-seeded conditions

To figure out this composition discrepancy, we performed thermodynamic calculations in the Au–Ga–In–P quaternary systems via combining the two Au–Ga–In and Ga–In–P thermodynamic databases [51, 52]. According to the pseudo-binary phase diagram (see Fig. 5a), there exists a miscibility gap in the ternary InGaP compound, which makes it challenging for the composition tunability in InGaP nanowires. Using thermo-calc software, we calculate the solidification of InGaP from supersaturated Au droplet to simulate the Au-seeded nanowire growth. According to the experiments, the temperature and indium composition range in the catalyst is 793–873 K and 50–80 at.%, respectively. The computed composition of InGaP during the addition of Ga in the droplet is shown in Fig. 5b. Thermodynamically, nanowire nucleation changes from InP to GaP when the Ga content is over 1 at.%. InGaP nanowires can only be formed if the Ga content is below 1 at.% while this conditions is hardly achievable in our experiments. Moreover, this trend is nearly independent of growth temperature and indium content in the catalyst. These calculations well explain the formation of GaP nanowires at different growth conditions. Also, it suggests the InGaP nanowire growth in our system is close to equilibrium conditions. Further driving force (∆*μ*) analysis in Fig. 5c explains such a sharp composition transformation. The driving force to form InP only slightly changes with variation of indium content and growth temperature in Au droplet. Instead, adding a small amount of Ga in the catalyst leads to a sharp change of driving force. The largely increased driving force causes the formation of GaP instead of InP, even though the gallium content in the droplet is over 10 times smaller than the indium. This is because thermodynamically, GaP is much more stable than InP. According to thermodynamic calculations, it is challenging to grow InGaP nanowires. Therefore, we suggest that growth conditions should be pushed to dynamics controlled region to form ternary nanowires [5]. Another approach is to grow InGaP nanowires within the selective area epitaxy approach [32]. Otherwise, Au catalyst should be replaced by another possible metal, or the nanowire should be grown without a catalyst [32]. We further calculated the situation for self-seeded InGaP nanowires in Fig. 5d. The driving force for InP nanowire formation is enhanced when compared with Au droplet. However, still, the driving force to form GaP is much larger than InP, suggesting that the self-catalyzed growth of InGaP nanowires via this method would still be challenging to realize composition control.

The above experimental observation and CALPHAD calculations suggest that thermodynamics is an essential factor in determining the III–V nanowire growth. Consequently, building a valid thermodynamic database, especially those including nanosize effect, and using the principle of CALPHAD approach can provide key thermodynamic information to guide the growth of III–V nanowires, including but not limited to composition and crystal structure.

## Conclusions

In conclusion, we successfully grow InP and GaP nanowires in a large area with a high density using a vacuum CVD method. PL and XPS analysis confirmed the formation of In_2_O_3_ at lower growth temperature and resulted in broad emission peak in the range of ~ 775 to 811 nm. Increasing the temperature helps avoid oxide formation and increase the purity of InP nanowires. Consequently, InP nanowires, grown at high-temperature, form a ZB twinning superlattice structure with a strong emission peak at room temperature. Moreover, we observed a sharp transition from InP to GaP nanowires by adding a small amount of Ga into the reactor, as confirmed by different characterization techniques. All the tested growth temperatures and the ratio of Ga/InP powder lead to GaP nanowire formation. Detailed EDX studies reveal a high indium content in the Au catalyst but not in the nanowire. A quaternary thermodynamic database (Au–In–Ga–P) is combined to calculate the solidification process. According to the calculations, only 1 at.% Ga in the Au catalyst is enough to transfer the nanowire growth from InP to GaP due to a sharp increase in nucleation driving force for GaP. Calculations also indicate this phenomenon is valid in a large growth condition range and also in self-nucleated InGaP nanowire growth, well explaining the observed growth fundamental. Therefore, we believe that thermodynamic calculation using a CALPHAD method helps guide III–V nanowire growth.

## Supplementary Information

**Additional file 1.** **Figure S1.** (a) HAADF image of a GaP nanowire. EDX mapping of (a). (b) Au, (c) Ga element and (d) In element.

## Data Availability

The authors declare that the data supporting the findings of this study are available within the article.

## Abbreviations

CALPHAD: The calculation of phase diagrams
MOCVD: Metal organic chemical vapor deposition
MBE: Molecular beam epitaxy
CVD: Chemical vapor deposition
SEM: Scanning electron microscope
TEM: Transmission electron microscope
EDS: Energy-dispersive spectrum
PL: Photoluminescence
VLS: Vapor–liquid–solid
LO: The longitudinal optical
TO: The traverse optical
ZB: Zincblende
WZ: Wurtzite
XPS: X-ray photoelectron spectroscopy
EDX: Energy-dispersive X-ray spectroscopy
HRTEM: High-resolution transmission electron microscope

## References

[1] Sun JM, Han MM, Gu Y, Yang ZX, Zeng HB (2018). Recent advances in group III–V nanowire infrared detectors. Adv Opt Mater.

[2] Yang ZX, Han N, Fang M, Lin H, Cheung HY, Yip S, Wang EJ, Hung T, Wong CY, Ho JC (2014). Surfactant-assisted chemical vapour deposition of high-performance small-diameter GaSb nanowires. Nat Commun.

[3] Shen L, Pun EYB, Ho JC (2017). Recent developments in III–V semiconducting nanowires for high-performance photodetectors. Mater Chem Front.

[4] Vukajlovic-Plestina J, Kim W, Ghisalberti L, Varnavides G, Tutuncuoglu G, Potts H, Friedl M, Guniat L, Carter WC, Dubrovskii VG, Fontcuberta IMA (2019). Fundamental aspects to localize self-catalyzed III–V nanowires on silicon. Nat Commun.

[5] Lim H, Young JL, Geisz JF, Friedman DJ, Deutsch TG, Yoon J (2019). High performance III–V photoelectrodes for solar water splitting via synergistically tailored structure and stoichiometry. Nat Commun.

[6] Yan X, Li B, Lin Q, Liu P, Luo Y, Lu Q, Zhang X, Ren X (2019). High performance transistors and photodetectors based on self-catalyzed zinc-blende InP nanowires. Appl Phys Lett.

[7] Zhang K, Ding J, Lou Z, Chai R, Zhong M, Shen G (2017). Heterostructured ZnS/InP nanowires for rigid/flexible ultraviolet photodetectors with enhanced performance. Nanoscale.

[8] Assali S, Zardo I, Plissard S, Kriegner D, Verheijen MA, Bauer G, Meijerink A, Belabbes A, Bechstedt F, Haverkort JE, Bakkers EP (2013). Direct band gap wurtzite gallium phosphide nanowires. Nano Lett.

[9] Liborius L, Bieniek J, Possberg A, Tegude F-J, Prost W, Poloczek A, Weimann N (2020). Tunneling-related leakage currents in coaxial GaAs/InGaP nanowire heterojunction bipolar transistors. Phys Status Solidi B.

[10] Gazibegovic S, Car D, Zhang H, Balk SC, Logan JA, de Moor MWA, Cassidy MC, Schmits R, Xu D, Wang G, Krogstrup P, Op Het Veld RLM, Zuo K, Vos Y, Shen J, Bouman D, Shojaei B, Pennachio D, Lee JS, van Veldhoven PJ, Koelling S, Verheijen MA, Kouwenhoven LP, Palmstrom CJ, Bakkers E (2017). Epitaxy of advanced nanowire quantum devices. Nature.

[11] Wagner RS, Ellis WC (1964). Vapor–liquid–solid mechanism of single crystal growth. Appl Phys Lett.

[12] Park JH, Pozuelo M, Setiawan BP, Chung CH (2016). Self-catalyzed growth and characterization of In(As)P nanowires on InP(111)B using metal-organic chemical vapor deposition. Nanoscale Res Lett.

[13] Khmissi H, Naji K, Hadj Alouane MH, Chauvin N, Bru-Chevallier C, Ilahi B, Patriarche G, Gendry M (2012). InAs/InP nanowires grown by catalyst assisted molecular beam epitaxy on silicon substrates. J Cryst Growth.

[14] Gao Q, Saxena D, Wang F, Fu L, Mokkapati S, Guo Y, Li L, Wong-Leung J, Caroff P, Tan HH (2014). Selective-area epitaxy of pure wurtzite InP nanowires: high quantum efficiency and room-temperature lasing. Nano Lett.

[15] Yuan X, Liu K, Skalsky S, Parkinson P, Jagadish C (2020). Carrier dynamics and recombination mechanisms in InP twinning superlattice nanowires. Opt Express.

[16] Yuan X, Guo Y, Caroff P, He J, Tan HH, Jagadish C (2017). Dopant-free twinning superlattice formation in InSb and InP nanowires. Phys Status Solidi RRL.

[17] Peng K, Jevtics D, Zhang F, Sterzl S, Damry D, Rothmann M, Guilhabert B, Strain M, Tan H, Herz L, Fu L, Dawson M, Hurtado A, Jagadish C, Johnston M (2020). Three-dimensional cross-nanowire networks recover full terahertz state. Science.

[18] Staudinger P, Mauthe S, Trivino NV, Reidt S, Moselund KE, Schmid H (2021). Wurtzite InP microdisks: from epitaxy to room-temperature lasing. Nanotechnology.

[19] Kornienko N, Gibson NA, Zhang H, Eaton SW, Yu Y, Aloni S, Leone SR, Yang P (2016). Growth and photoelectrochemical energy conversion of wurtzite indium phosphide nanowire arrays. ACS Nano.

[20] Sun J, Yin Y, Han M, Yang ZX, Lan C, Liu L, Wang Y, Han N, Shen L, Wu X, Ho JC (2018). Nonpolar-oriented wurtzite InP nanowires with electron mobility approaching the theoretical limit. ACS Nano.

[21] Janissen R, Sahoo PK, Santos CA, da Silva AM, von Zuben AAG, Souto DEP, Costa ADT, Celedon P, Zanchin NIT, Almeida DB, Oliveira DS, Kubota LT, Cesar CL, Souza AP, Cotta MA (2017). InP nanowire biosensor with tailored biofunctionalization: ultrasensitive and highly selective disease biomarker detection. Nano Lett.

[22] Song T, Lee S-T, Sun B (2012). Silicon nanowires for photovoltaic applications: the progress and challenge. Nano Energy.

[23] Standing A, Assali S, Gao L, Verheijen MA, van Dam D, Cui Y, Notten PH, Haverkort JE, Bakkers EP (2015). Efficient water reduction with gallium phosphide nanowires. Nat Commun.

[24] Hui AT, Wang F, Han N, Yip S, Xiu F, Hou JJ, Yen Y-T, Hung T, Chueh Y-L, Ho JC (2012). High-performance indium phosphide nanowires synthesized on amorphous substrates: from formation mechanism to optical and electrical transport measurements. J Mater Chem.

[25] Zheng D, Wang J, Hu W, Liao L, Fang H, Guo N, Wang P, Gong F, Wang X, Fan Z, Wu X, Meng X, Chen X, Lu W (2016). When nanowires meet ultrahigh ferroelectric field-high-performance full-depleted nanowire photodetectors. Nano Lett.

[26] Gagliano L, Kruijsse M, Schefold JDD, Belabbes A, Verheijen MA, Meuret S, Koelling S, Polman A, Bechstedt F, Haverkort JEM, Bakkers EPAM (2018). Efficient green emission from wurtzite Al_x_In_1–x_P Nanowires. Nano Lett.

[27] Wilson DJ, Schneider K, Hönl S, Anderson M, Baumgartner Y, Czornomaz L, Kippenberg TJ, Seidler P (2020). Integrated gallium phosphide nonlinear photonics. Nat Photonics.

[28] Malizia M, Seger B, Chorkendorff I, Vesborg PCK (2014). Formation of a p–n heterojunction on GaP photocathodes for H2 production providing an open-circuit voltage of 710 mV. J Mater Chem A.

[29] Gao Q, Saxena D, Wang F, Fu L, Mokkapati S, Guo Y, Li L, Wong-Leung J, Caroff P, Tan HH, Jagadish C (2014). Selective-area epitaxy of pure wurtzite InP nanowires: high quantum efficiency and room-temperature lasing. Nano Lett.

[30] Wang Y, Hegde M, Chen S, Yin PV, Radovanovic P (2018). Control of the spontaneous formation of oxide overlayers on GaP nanowires grown by physical vapor deposition. AIMS Mater Sci.

[31] Wallentin J, Barrutia Poncela L, Jansson AM, Mergenthaler K, Ek M, Jacobsson D, Reine Wallenberg L, Deppert K, Samuelson L, Hessman D, Borgström MT (2012). Single GaInP nanowire p-i-n junctions near the direct to indirect bandgap crossover point. Appl Phys Lett.

[32] Berg A, Caroff P, Shahid N, Lockrey MN, Yuan X, Borgström MT, Tan HH, Jagadish C (2016). Growth and optical properties of In_x_ Ga_1−x_ P nanowires synthesized by selective-area epitaxy. Nano Res.

[33] Fakhr A, Haddara YM, Lapierre RR (2010). Dependence of InGaP nanowire morphology and structure on molecular beam epitaxy growth conditions. Nanotechnology.

[34] Du Y, Sundman B (2017). Thermophysical properties: key input for ICME and MG. J Phase Equilib Diffus.

[35] Ghasemi M, Zanolli Z, Stankovski M, Johansson J (2015). Size- and shape-dependent phase diagram of In–Sb nano-alloys. Nanoscale.

[36] Ghasemi M, Johansson J (2017). Phase diagrams for understanding gold-seeded growth of GaAs and InAs nanowires. J Phys D Appl Phys.

[37] Leshchenko ED, Ghasemi M, Dubrovskii VG, Johansson J (2018). Nucleation-limited composition of ternary III–V nanowires forming from quaternary gold based liquid alloys. CrystEngComm.

[38] Halder NN, Kelrich A, Cohen S, Ritter D (2018). Controlled axial and radial growth of InP nanowires by metal-organic molecular beam epitaxy using the selective-area vapour–liquid–solid approach. Nanotechnology.

[39] Rizal U, Swain BP, Swain BS (2016) Gallium phosphide nanowires for optoelectronic devices. IEEE 16177729

[40] Zhang J, Fryauf DM, Norris KJ, Wei M, Diaz Leon JJ, Kobayashi NP (2014). Raman spectroscopy of indium phosphide nanowire networks coated with gold clusters. J Mater Sci Mater Electron.

[41] Gong K, Sun C, Xiong B, Han Y, Hao Z, Wang J, Wang L, Li H (2017). Oxides formation on hydrophilic bonding interface in plasma-assisted InP/Al_2_O_3_/SOI direct wafer bonding. AIP Adv.

[42] Lin J, You T, Jin T, Liang H, Wan W, Huang H, Zhou M, Mu F, Yan Y, Huang K, Zhao X, Zhang J, Wang S, Gao P, Ou X (2020). Wafer-scale heterogeneous integration InP on trenched Si with a bubble-free interface. APL Mater.

[43] Yadav K, Mehta BR, Singh JP (2015). Presence of metal-oxide interface enhanced photoluminescence from In–In_2_O_3_ core–shell nanorods. RSC Adv.

[44] Tomita T, Yamashita K, Hayafuji Y, Adachi H (2005). The origin of n-type conductivity in undoped In_2_O_3_. Appl Phys Lett.

[45] Patzke GR, Kontic R, Shiolashvili Z, Makhatadze N, Jishiashvili D (2012). Hydrazine-assisted formation of indium phosphide (InP)-based nanowires and core–shell composites. Mater Basel.

[46] Park K, Lee JA, Im HS, Jung CS, Kim HS, Park J, Lee CL (2014). GaP–ZnS pseudobinary alloy nanowires. Nano Lett.

[47] Clarke MT, Viscomi FN, Chamberlain TW, Hondow N, Adawi AM, Sturge J, Erwin SC, Bouillard J-SG, Tamang S, Stasiuk GJ (2019). Synthesis of super bright indium phosphide colloidal quantum dots through thermal diffusion. Commun Chem.

[48] Nguyen Thanh T, Robert C, Guo W, Létoublon A, Cornet C, Elias G, Ponchet A, Rohel T, Bertru N, Balocchi A, Durand O, Micha JS, Perrin M, Loualiche S, Marie XL, Corre A (2012). Structural and optical analyses of GaP/Si and (GaAsPN/GaPN)/GaP/Si nanolayers for integrated photonics on silicon. J Appl Phys.

[49] Shang L, Song L, Wang Y, Cai R, Liu L, Wang F (2018). Formation mechanisms of InGaAs nanowires produced by a solid-source two-step chemical vapor deposition. Nanoscale Res Lett.

[50] Yuan X, Yang J, He J, Tan HH, Jagadish C (2018). Role of surface energy in nanowire growth. J Phys D Appl Phys.

[51] Kim H, Farrell AC, Senanayake P, Lee WJ, Huffaker DL (2016). Monolithically integrated InGaAs nanowires on 3D structured silicon-on-insulator as a new platform for full optical links. Nano Lett.

[52] Ghasemi M, Sundman B, Fries SG, Johansson J (2014). The thermodynamic assessment of the Au–In–Ga system. J Alloys Compd.

