# Supplementary material for: Thermodynamics Controlled Sharp Transformation from InP to GaP Nanowires via Introducing Trace Amount of Gallium
Source: Nanoscale Res Lett. 2021 Mar 20;16:49. doi: 10.1186/s11671-021-03505-2 (PMC7981363; doi:10.1186/s11671-021-03505-2)
Supplement: Supplementary file 1 — Additional file 1. Figure S1. (a) HAADF image of a GaP nanowire. EDX mapping of (a). (b) Au, (c) Ga element and (d) In element. [file 11671_2021_3505_MOESM1_ESM.docx]

**Supporting information**

**Thermodynamics controlled sharp transformation from InP to GaP nanowires via introducing trace amount of Gallium.**

Zhenzhen Tian^1^, Xiaoming Yuan^1^*, Ziran Zhang^1^, Wuao Jia^1^, Jian Zhou^2^, Han Huang^1^, Jianqiao Meng^1^, Jun He^1^*, Yong Du^3^
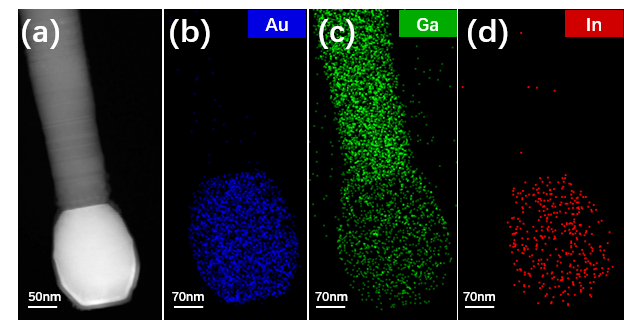


Figure S1. (a) HAADF image of a GaP nanowire. EDX mapping of (a). (b) Au, (c) Ga element and (d) In element. From the brightness contrast and the EDX mapping, it seems there exists a Au-rich layer at the top surface .
